# Supervised machine learning to support the diagnosis of bacterial infection in the context of COVID-19

**DOI:** 10.1093/jacamr/dlab002

**Published:** 2021-02-03

**Authors:** Timothy M Rawson, Bernard Hernandez, Richard C Wilson, Damien Ming, Pau Herrero, Nisha Ranganathan, Keira Skolimowska, Mark Gilchrist, Giovanni Satta, Pantelis Georgiou, Alison H Holmes

**Affiliations:** 1 National Institute for Health Research Health Protection Research Unit in Healthcare Associated Infections and Antimicrobial Resistance, Imperial College London, Hammersmith Campus, Du Cane Road, London W12 0NN, UK; 2 Centre for Antimicrobial Optimisation, Hammersmith Hospital, Imperial College London, Du Cane Road, London W12 0NN, UK; 3 Imperial College Healthcare NHS Trust, Hammersmith Hospital, Du Cane Road, London W12 0HS, UK; 4 Centre for Bio-inspired Technology, Department of Electrical and Electronic Engineering, Imperial College London, South Kensington Campus, London SW7 2AZ, UK

## Abstract

**Background:**

Bacterial infection has been challenging to diagnose in patients with COVID-19. We developed and evaluated supervised machine learning algorithms to support the diagnosis of secondary bacterial infection in hospitalized patients during the COVID-19 pandemic.

**Methods:**

Inpatient data at three London hospitals for the first COVD-19 wave in March and April 2020 were extracted. Demographic, blood test and microbiology data for individuals with and without SARS-CoV-2-positive PCR were obtained. A Gaussian Naive Bayes, Support Vector Machine (SVM) and Artificial Neural Network were trained and compared using the area under the receiver operating characteristic curve (AUCROC). The best performing algorithm (SVM with 21 blood test variables) was prospectively piloted in July 2020. AUCROC was calculated for the prediction of a positive microbiological sample within 48 h of admission.

**Results:**

A total of 15 599 daily blood profiles for 1186 individual patients were identified to train the algorithms; 771/1186 (65%) individuals were SARS-CoV-2 PCR positive. Clinically significant microbiology results were present for 166/1186 (14%) patients during admission. An SVM algorithm trained with 21 routine blood test variables and over 8000 individual profiles had the best performance. AUCROC was 0.913, sensitivity 0.801 and specificity 0.890. Prospective testing on 54 patients on admission (28/54, 52% SARS-CoV-2 PCR positive) demonstrated an AUCROC of 0.960 (95% CI: 0.90–1.00).

**Conclusions:**

An SVM using 21 routine blood test variables had excellent performance at inferring the likelihood of positive microbiology. Further prospective evaluation of the algorithms ability to support decision making for the diagnosis of bacterial infection in COVID-19 cohorts is underway.

## Introduction

The emergence of SARS-CoV-2 has created new challenges for clinical practice. One challenge is identifying patients with bacterial infection.^1–3^ In COVID-19 patients, rates of detected bacterial or fungal infection appear to be low. In contrast, the use of empirical antimicrobial therapy has been widely observed.^1–3^

Bacterial infection is challenging to diagnose in patients with COVID-19 as many of the traditional clinical indicators are also affected by infection with the SARS-CoV-2 virus. These include fever, raised inflammatory markers such as C-reactive protein (CRP) and abnormal chest radiographs.

In patients admitted to hospital with COVID-19, WHO has recommended that empirical antimicrobial therapy be avoided in patients with mild to moderate disease unless clear evidence of bacterial coinfection is present.^4^ This is due to concerns about the impact of excessive antimicrobial prescribing in COVID-19 on antimicrobial resistance.^4–6^

We previously reported on the use of supervised machine learning to support the prediction of bacterial infection in patients admitted to hospital.^7^^,^^8^ A Support Vector Machine (SVM) using six routinely available blood test results was able to infer the likelihood of an individual patient having a positive microbiological sample. This was used to infer the likelihood of the patient developing an infection. The algorithm was trained with six independent variables and was deployed locally during the COVID-19 pandemic. It was observed that in COVID-19 patients, using only six variables reduced the predictive capability of the current algorithm. This is likely driven by COVID-19 having a significant influence on these variables, reducing the discriminatory ability of the algorithm when using only six independent blood test results. Within this study, we explored the development of supervised machine learning algorithms that can predict bacterial infection in patients admitted to hospital during the COVID-19 pandemic using an expanded range of routinely available blood test results. The algorithm was designed and tested on patients both with and without COVID-19 and aimed to provide optimal predictive capabilities using routinely available blood test results.

## Methods

### Study setting

This study aimed to develop, validate and compare supervised machine learning algorithms to support the diagnosis of bacterial infection in all patients admitted to hospital during the COVID-19 pandemic. The study was performed at three hospitals in North West London, serving a population of over 2 million individuals.

### Participants

Patients included in the development and evaluation of the algorithms were admitted during the initial surge in UK cases between March and April 2020. All patients testing positive for SARS-CoV-2 using nasopharyngeal swab PCR during this time were included. For algorithm development and evaluation, we aimed to include SARS-CoV-2 PCR-positive and -negative individuals admitted during this period. To facilitate this, we aimed to achieve a 2:1 ratio of SARS-CoV-2-positive and -negative cases respectively of similar age, gender and microbiology. Participant information was anonymously extracted from the patient electronic health record system, including demographic, clinical, physiological, biochemical and microbiological results. Clinically significant microbiology results were determined by two infectious disease and microbiology experts independently of each other.

For prospective pilot testing of the algorithm, patients admitted between July and August 2020 were eligible for inclusion. Individuals who were reviewed by an infectious disease specialist were identified and prospectively included in the algorithm. This followed an opportunistic format with individuals reviewed by the specialist on admission eligible for inclusion during this period.

### Algorithm development, cross validation and external piloting

Algorithm development, data handling and cross validation were performed using Scikit-learn (0.23.1) and Pandas (1.0.3). The algorithms are deployed within a CE-marked clinical decision support system, EPIC IMPOC.^9^ Several supervised machine learning algorithms were trained and compared during this study. Algorithms tested were based on prior experience of developing machine learning tools for infection inference.^7^^,^^8^ Algorithms used were Gaussian Naive Bayes (GNB), SVM and Artificial Neural Network (ANN) algorithms. For this study GNB, SVM and ANN using either 6 or 21 routinely available blood test results were compared. This included the SVM using six blood test variables previously evaluated.^7^^,^^8^ Further information on blood test result selection, the training process and the algorithms is provided in Appendix S1 (available as Supplementary data at *JAC-AMR* Online).

### Algorithm comparison and statistical analysis

Descriptive analysis using χ^2^ test for non-parametric data were performed. For identification of highly correlated blood test results, Pearson’s correlation coefficient was used with values between 0.7 and 1.0 deemed to have a strong linear correlation. For training and validation, 10-fold stratified cross validation was performed. Algorithms were compared using the area under the receiver operating characteristic curve (AUCROC), sensitivity, specificity and geometric mean. For prospective piloting, AUCROC was used. Different probability cut-offs were evaluated using the AUCROC to select a cut-off that would provide optimal sensitivity and specificity.

### Ethics

This study was registered locally within the institution as part of a service evaluation (Imperial College Healthcare NHS Trust ref. 458) using routinely available, anonymized healthcare data. Ethical approval was not required.

### Availability of data and material

The datasets used and/or analysed during the current study are available from the corresponding author on reasonable request where not presented in the manuscript or figure.

## Results

### Participants

Between March and April 2020, 1186 patients were identified. These included 771/1186 (65%) SARS-CoV-2 PCR-positive patients and 415/1186 (35%) negative patients. The median age of the cohort was 65 (IQR: 48–78) years. Most patients were male (624/1186; 53%). Median length of stay for the cohort was 8 (IQR: 4–17) days.

In total, 166/1186 (14%) individual patients had a positive, clinically relevant, microbiological culture result during their admission. Of those with positive SARS-CoV-2 PCR, 104/771 (13%) had clinically relevant microbiology during their admission. This was compared with 62/415 (15%) in SARS-CoV-2-negative individuals (*P *=* *0.55).

### Selection of blood test variables for inclusion

For 1186 individual patients, 15 599 daily patient blood profiles were extracted and linked to either positive clinically significant or no (unavailable or non-significant) microbiology on individual days. Figure S1 outlines the frequency at which individual blood test parameters were requested in daily patient profiles. In total, 374 individual blood and biochemical tests were evaluated, with 27/374 (7%) found to be frequently available in over 8000 of the daily profiles. These 27 variables were explored with highly correlated variables removed (Figure S2). This left 21 variables included as input variables to train the algorithms.

### Algorithm performance

**Figure 1.**
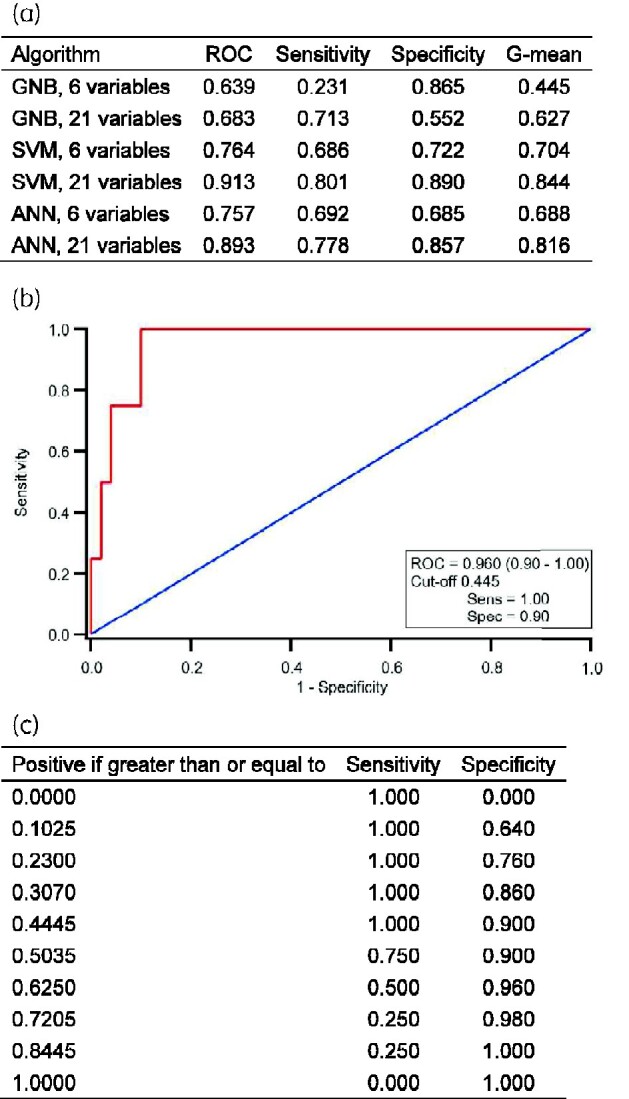
Comparison of supervised machine learning algorithms and summary of prospective evaluation of an SVM with 21 routine variables. (a) Comparison of supervised machine learning algorithms for the prediction of positive microbiology during COVID-19 pandemic. (b) Receiver operator characteristics (ROC) for an SVM used to predict positive microbiology within 48 h of admission to hospital. From a prospective pilot of 54 patients admitted to hospital between July and August 2020; 28/54 (52%) were SARS-CoV-2 PCR positive. (c) Summary of sensitivity and specificity from the ROC for an SVM using 21 blood test results to predict positive microbiology when different cut-off values are selected. ROC, area under the curve receiver operator characteristic; G-mean, geometric mean.

Figure 1 summarizes the results of 10-fold stratified cross validation for GNB, SVM and ANN algorithms trained with the 6 original input parameters used in prior work and 21 commonly available blood tests identified as part of this study.

Overall, SVM with 21 variables was found to have the best performance with an AUCROC 0.913, sensitivity 0.801 and specificity 0.890 for all individuals. Inclusion of 21 versus 6 blood test parameters improved the performance of all three supervised machine learning tools. Probability density distributions of SARS-CoV-2 and non-SARS-CoV-2 patients demonstrated a similar performance (Figure S3).

### Prospective pilot

Between July and August 2020, routine blood test data from 54 patients were included in the chosen SVM algorithm. Of those admitted, 28/54 (52%) were SARS-CoV-2 PCR positive. Only 4/54 (7%) had positive microbiology within 48 h of admission. Of those that were SARS-CoV-2 PCR positive, 2/28 (7%) had a positive microbiological sample. The AUCROC using admission blood tests was 0.960 (0.91–1.00). A cut-off probability for the SVM of 0.445 was found to give a sensitivity of 100% and specificity of 90% (Figure 1).

## Discussion

We demonstrate that a supervised machine learning algorithm, trained using 21 routinely available blood test results from patients admitted to hospital during the COVID-19 pandemic, can accurately predict the likelihood of positive microbiology in future patients. An SVM with 21 variables demonstrated the best performance and was superior to a previously validated algorithm using six blood test parameters tested prospectively on patients admitted to hospital, prior to the pandemic.^8^

COVID-19 has posed many significant challenges to human health. The long-term impact of COVID-19 on inappropriate prescribing and thus antimicrobial resistance is a concern.^5^^,^^6^ With reported low rates of bacterial infection in COVID-19 patients, high rates of antimicrobial prescribing and challenges identifying those at risk of coinfection, urgent mechanisms to support antimicrobial decision making are required. The use of machine learning to support decision making in infection management has been previously explored, but limited evidence is available to support its use in management of bacterial infection in COVID-19.^10^^,^^11^ Prospective implementation and evaluation of this decision support tool will now be undertaken to evaluate its impact in clinical practice to support decision making for infection management.

This study had several limitations. First, this algorithm predicts the likelihood of positive clinically significant microbiology. This is used to infer the likelihood of infection. This is reliant on clinicians requesting and performing of appropriate microbiological sampling in clinical practice and on good microbiological yield from samples. It also did not include bacterial infection specific biomarkers, such as procalcitonin. Second, this study only looked to compare algorithm performance and pilot the chosen algorithm on a small selection of patients admitted to hospital. Within the pilot, there were only a small number of individuals (4/54; 7%) who had positive microbiology. For prospective work, admission blood tests were used to try and avoid potential confounders, such as the influence of antibiotic therapy or other procedures/interventions that may influence blood test results. Future prospective work is required to evaluate the use of this algorithm longitudinally and understand its influence on clinician antibiotic prescribing behaviour in practice.

### Conclusions

An SVM with 21 blood test variables demonstrated excellent performance when compared against an ANN and GBM algorithm at predicting the likelihood of an individual having positive microbiology in a cohort of patients admitted to hospital during the COVID-19 pandemic. Adoption of decision support tools to support diagnosis of bacterial coinfection and secondary infection may help reduce unnecessary antimicrobial prescribing in COVID-19 and thus prevent the propagation of antimicrobial resistance.

## Supplementary Material

dlab002_Supplementary_Data
