# Supplementary material for: Supervised machine learning to support the diagnosis of bacterial infection in the context of COVID-19
Source: JAC Antimicrob Resist. 2021 Feb 3;3(1):dlab002. doi: 10.1093/jacamr/dlab002 (PMC7928888; doi:10.1093/jacamr/dlab002)
Supplement: dlab002_Supplementary_Data [file dlab002_supplementary_data.docx]

**Supplementary data**

**Figure S1.** Summary of daily blood test result availability for individuals included within the training dataset for this study.


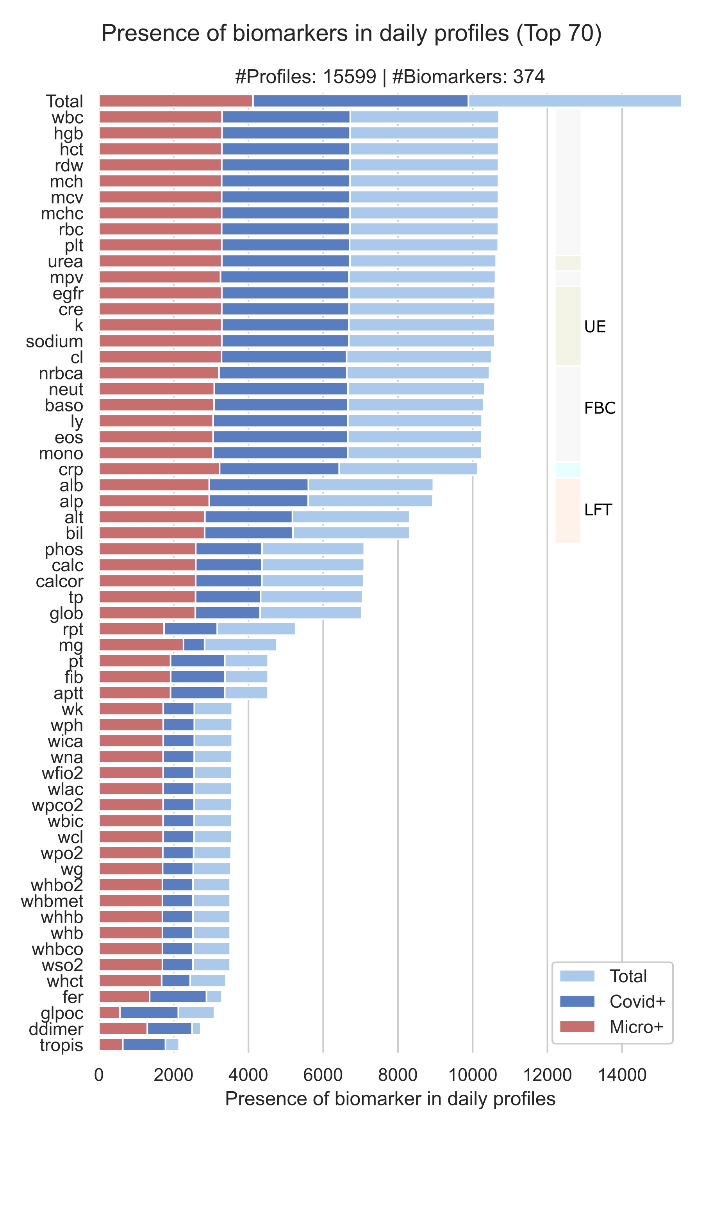


**Legend:** The dataset considered contained 15599 daily profiles with a total of 374 different blood tests. This figure shows the prevalence for the top 70 blood tests including the total amount of daily profiles (light blue), the proportion of profiles from patients with diagnosed covid-19 (dark blue) and the proportion of profiles from patients with a positive microbiology sample (red).

The chemistry panels to determine the groups of tests that are routinely requested together have been specified for those blood tests considered in this study: (i) UE: Urea and Electrolyte panel, (ii) FBC: Full blood count and (iii) LFT: Liver Function Test. Note that some blood tests are requested independently and do not correspond to any chemical panel such as C-Reactive Protein (CRP).

**Figure S2.** Common blood test results available in daily patient profiles and their correlation

| Code | Description | Unit |
| --- | --- | --- |
| alb* | Albumin | g/L |
| alp* | Alkaline Phosphatase | U/L |
| alt* | Alanine Transaminase | U/L |
| baso* | Basophils | 10*9/L |
| bil* | Bilirubin | umol/L |
| cl* | Chloride | mmol/L |
| cre* | Creatinine | umol/L |
| crp* | C-Reactive Protein | mg/L |
| egfr* | Estimated GFR | mL/min/1.73m2 |
| eos* | Eosinophils | 10*9/L |
| hct | Haematocrit | L/L |
| hgb | Haemoglobin | g/L |
| k* | Potassium | mmol/L |
| ly* | Lymphocytes | 10*9/L |
| mch* | Mean cell haemoglobin level | pg |
| Mchc | Mean cell haemoglobin conc | g/L |
| mcv | Mean cell volume | fL |
| mono* | Monocytes | 10*9/L |
| mpv* | Mean platelet volume | fL |
| neut | Neutrophils | 10*9/L |
| nrbca* | Nucleated RBCs | 10*9/L |
| plt* | Platelets | 10*9/L |
| rbc* | Red blood cell count | 10*12/L |
| rdw* | Red blood cell distribution width | % |
| sodium | Sodium | mmol/L |
| urea* | Urea level | mmol/L |
| wbc* | White blood cell count | 10*9/L |


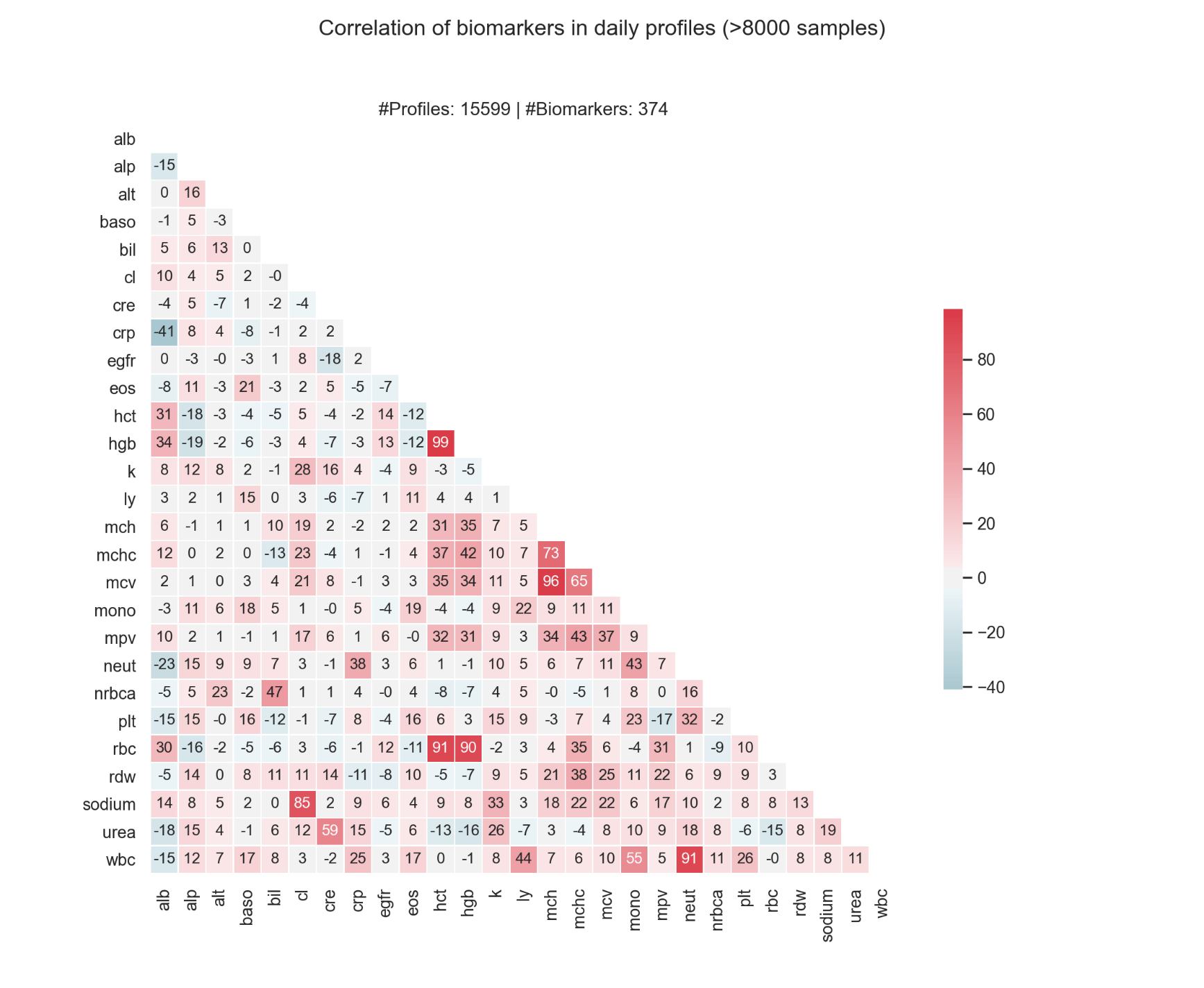


**Legend:** *Left:* Table of 27 blood test results investigated for inclusion in the supervised machine learning algorithm. * Indicates blood test results included in the algorithm. *Right*: Correlation matrix of 27 blood tests considered for use the machine learning algorithm. The correlation matrix was computed using the Pearson correlation coefficient which measures the linear relationship between two variables. Coefficients whose absolute value is within [0.7, 1.0] indicate strong linear relationship and therefore the corresponding blood tests have been considered redundant and were discarded.

**Figure S3.** Probability distribution for predicting positive microbiology using a Support Vector Machine with 21 routinely available input variables.


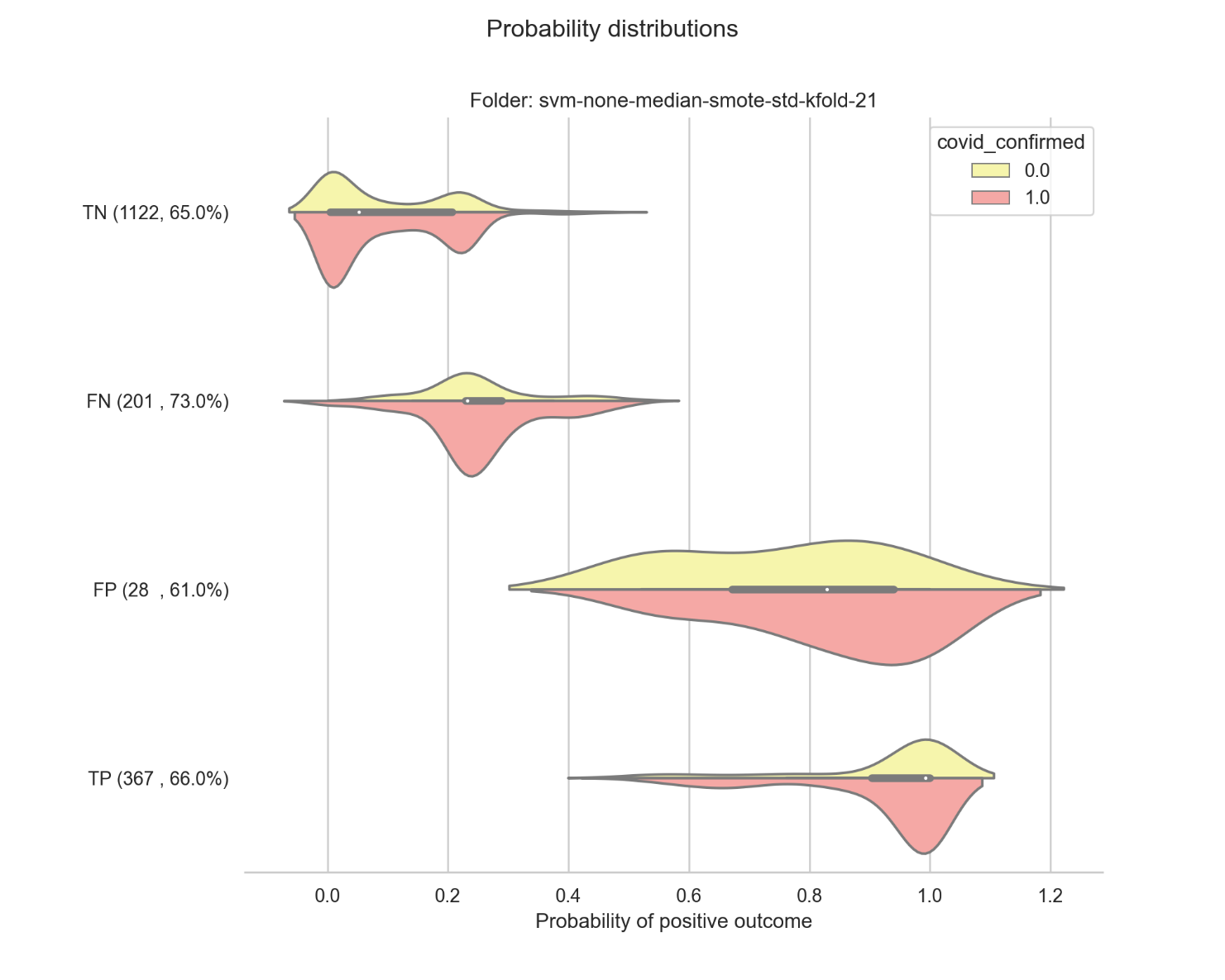


**True negative**

**False negative**

**False positive**

**True positive**

**Legend:** The density distributions for each type of classification is shown in this figure for both COVID confirmed (red) and COVID negative (yellow) patients. Distributions for each type of classification (true positive, true negative, false positive and false negative) are shown. It appears that extreme probabilities generally correspond to correct predictions.

**Appendix S1.** Description of algorithms and training methods applied.

**Gaussian Naïve Bayes (GNB)**

GNB is based on applying Bayes’ theorem with the assumption of independence between every pair of features. The likelihood function for each feature is assumed to be Gaussian and despite this simplifying assumption, it has worked quite well in many real-world situations (e.g. spam filtering). In addition, they require a small amount of training data to estimate the necessary parameters, are extremely fast compared to more sophisticated methods and the generated models can perform online updates.

**Support vector machine (SVM)**

SVM uses a kernel function to transform the training samples to a new space with higher dimensionality. The boundary found in the high dimensional space is the hyperplane which maximizes the distance between classes (i.e. maximum margin hyperplane) and can have a non-linear shape in the original data space. It employs the principle of Structural Risk Minimization to generalize better than conventional machine learning methods which employ Empirical Risk Minimization. Though SVMs do not directly provide probability estimates, they may be calculated in the binary case using Platt scaling; that is, logistic regression on the SVM’s scores.

**Artificial Neural Network (ANN)**

An ANN contains a collection of connected nodes, called artificial neurons. These artificial neurons are modelled on a simplified structure of the human brain. Each connection, like synapses of the brain can transmit signals between neurons. The signal at each neuron is a specific number that can then be processed using a non-linear function of the sum of all inputs to that neuron. Connections to the neuron are called edges. Both neurons and edges typically are weighted, with this weight adjusted at the ANN learns. Typically neurons are aggregated in to layers within the ANN with each layer performing different transformations on their inputs. Signals traverse from the first layer to the final layer where an output is generated.

**Blood test result selection**

The hospital designates chemistry panels to determine groups of tests that are routinely ordered together to determine a person’s general health status. The frequency to which this chemistry panels appear in the daily profiles are outlined in the **Figure S1**. The blood tests considered in our previous studies [7, 8] were the Liver Function Test (LFT panel), inflammatory markers (CRP) and white blood cell counts (WBC). In this study, we also included biomarkers appearing with the same or higher frequency on the daily profiles; that is, the Full Blood Count (FBC) and the Urea and Electrolyte (UE) panels. This trade maximizes the number of different blood tests considered while maintaining the total number of daily profiles.

The correlation matrix in **Figure S2** was computed using the Pearson correlation coefficient which measures the linear relationship between two variables. Coefficients whose absolute value is within [0.7, 1.0] indicate strong linear relationship and therefore the corresponding blood tests have been considered redundant and were discarded.

**Algorithm training**

Binary outcome data for individual patients was the presence of positive microbiology during admission (present or absent). The likelihood of positive microbiology is used to infer the presence of infection as previously described.^7,8^ For each algorithm, input vectors from the most commonly requested blood tests were included, with different combinations of blood tests results selected. Common blood tests available in the majority of patient profiles were also explored, with highly correlated tests removed before also being included within the algorithm.

The process followed for data cleaning, outlier detection, model training and model evaluation is fully described in [7].

Summarising this; outliers were removed using the inter-quartile range rule and the complete profiles (those for which all considered blood tests were available) were divided into Cross-Validation Set (CVS) and Hold-out Set (HOS). Grid-Search Ten-Fold Stratified Cross-Validation was performed on the CVS and all the models and their performance on the HOS were saved.

We acknowledge the complexity of dealing with missing data and the repercussion that the imputation technique might have on the predictions (e.g., median/mean imputation or inference of missing values based on other available blood tests in a round-robin fashion). For this reason, the algorithms are trained and retrospectively evaluated using exclusively complete daily profiles; that is, daily profiles with the 6 or 21 selected blood test results available, respectively.

Regarding the parameter tuning, the prior probabilities for GNB were adjusted according to the prevalence of the classes in the data and therefore no grid search was needed.

For the SVM we evaluated linear, polynomic and radial basis function (RBF) kernels. From this, the RBF provided the best results. The kernel coefficient ‘gamma’ of 0.1 consistently provided higher ROCs with balanced sensitivity and specificity. On the contrary, while a ‘gamma’ of 1.0 provided the highest ROCs (~0.95) the balance between sensitivity (~0.53) and specificity (~0.98) was poor.

For the ANN we evaluated the Logistic and ReLU activation functions, but they did not significantly affect the performance. On the contrary, the number of hidden layers clearly affected the performance. One single hidden layer with 50 nodes performed consistently better than multiple hidden layers. Regarding the penalty parameter ‘alpha’, there was not a significant difference in the range [0.0001, 0.1] however an ‘alpha’ value of 1 consistently provided slightly worst results. Weconsidered the top 35 ANN models within this process.The configuration parameters of the grids were iteratively modified after investigating the results. The ones shown below correspond to the last iteration.

*svm_grid = {*

*'C': [0.1, 1.0, 0.01],*

*'kernel': ['rbf'],*

*'gamma': [0.1, 1.0, 0.01],*

*'probability': [True],*

*'max_iter': [-1],*

*}*

*# Artificial neural network (ANN)*

*ann_grid = {*

*'hidden_layer_sizes': [(1,), (10,), (50,), (5, 5), (10, 10), (5, 5, 5)],*

*'activation': ['logistic', 'relu'],*

*'solver': ['adam'],*

*'alpha': [1., 0.1, 0.0001],*

*'batch_size': ['auto'],*

*'learning_rate': ['constant'],*

*'learning_rate_init': [0.001],*

*'power_t': [0.5],*

*'max_iter': [1000],*

*'tol': [1e-4],*

*'warm_start': [False],*

*'momentum': [0.9],*

*}*
